# Metabolic syndrome affects narrow-band UVB phototherapy response in patients with psoriasis

**DOI:** 10.1097/MD.0000000000008677

**Published:** 2017-12-15

**Authors:** Wang Rui, Ding Xiangyu, Xie Fang, Gong Long, Yang Yi, Wang Wenjuan, Hao Tian, Zhang Xiaoning, Zhou Yong, Fan Jianfeng, Li Hengjin, Li Chengxin

**Affiliations:** Department of Dermatology, Chinese PLA General Hospital, Medical College of Chinese PLA, Beijing, China.

**Keywords:** metabolic syndrome, NB-UVB, psoriasis, systemic inflammation, treatment

## Abstract

Psoriasis is a chronic inflammatory skin disease. Metabolic syndrome (MS) is a combination of central obesity, dyslipidemia, glucose intolerance, and elevated blood pressure. Many epidemiological surveys have revealed the association of psoriasis with MS. Narrowband ultraviolet radiation b (NB-UVB) is an effective and widely used treatment for psoriasis. The purpose of this study was to investigate whether the presence of MS in patient with psoriasis affects NB-UVB treatment and whether this syndrome correlates with systemic inflammation.

From June 2016 to December 2016, 243 adults with a diagnosis of psoriasis vulgaris eligible to treatment with NB-UVB were admitted to the phototherapy unit of Dermatology department, Chinese PLA General Hospital. Fifty-five included patients were grouped based on the presence of MS. They accepted the treatment of NB-UVB and the following data were collected: serum levels of IL-17 (interleukin), TNF-α (tumor necrosis factor) and IL-6, Psoriasis Area and Severity Index (PASI) scores before and after 10 sections of NB-UVB treatment.

Significant PASI improvement was observed in psoriatic patients without MS after 10 sections of phototherapy, while patients with MS showed a less improvement (*P* < .001). There was statistically significant difference in percentage of patients achieving 50% reduction in PASI scores between the 2 groups (*P* < .05). Multivariate logistic regression analysis showed MS was an independent factor that affecting the treatment of NB-UVB (*P* < .05). Psoriatic patients with MS showed a much less reduction of IL-17 and IL-6 before and after 10 sections of NB-UVB treatment respectively than patients without MS (*P* < .05).

Psoriatic patients with MS have poorer improvement in comparison those without MS using NB-UVB treatment. MS was an independent factor that affecting the treatment of NB-UVB. In addition, psoriatic patients with MS showed a much less reduction of systemic biomarkers (interleukin—IL-17, TNF-α, IL-6) than patients without MS. Namely, they may need a longer course of treatment to achieve improved skin lesions.

## 1 Introduction

Psoriasis is a chronic inflammatory skin disease with a genetic basis characterized by epidermal hyperproliferation, abnormal keratinocyte differentiation, T-lymphocyte infiltration, and increased expression of cytokines, which results in the formation of inflamed plaques.^[1,2]^ Metabolic syndrome (MS) is a combination of central obesity, dyslipidemia, glucose intolerance, and elevated blood pressure (BP).^[3]^ Many epidemiological surveys have revealed the association of psoriasis with MS, and the greater the severity of psoriasis, the stronger this association is shown.^[1,4]^ In addition, improvement of MS ameliorated skin lesion of psoriasis greatly. These evidences suggest that the presence of MS may promote the progression of psoriasis.^[1–4]^

Phototherapy is a standard treatment of psoriasis.^[4]^ Among the different modalities of treatment, narrow-band ultraviolet B phototherapy (NB-UVB) with TL01 lamps, emitting an electromagnetic spectrum centered in 311 nm, is the most widespread option.^[4,5]^ Its mode of action involves direct cytotoxicity against Th1 lymphocytes, dendritic cells, Langerhans cells, keratinocyte apoptosis, and upregulation of Treg lymphocytes.^[5]^ The response to NB-UVB varies depending on many factors. As a general rule, a worse response is expectable with big size, infiltrated psoriasis plaques, low phototype, and psychological stress.^[5–7]^ A better response is achieved in female patients and in patients with lower body mass index (BMI).^[8]^ However, the response of psoriasis patients with MS to UVB treatment has been relatively underexplored.

The purpose of this study was to investigate whether the presence of MS affects NB-UVB treatment in patient with psoriasis and whether this syndrome correlates with systemic inflammation.

## 2 Materials and methods

### 2.1 Eligible patient

From June 1, 2016 to December 20, 2016, 243 adult with a diagnosis of psoriasis vulgaris eligible to treatment with NB-UVB were admitted to the phototherapy unit of Dermatology Department, Chinese PLA General Hospital and 120 eligible patients accepted to participate. Among eligible patients we excluded those with psoriatic arthritis and those having taken systemic treatment in 2 months, finally remaining 55 cases. All patients provided a written informed consent to participate to the study and the Ethics Committee of Chinese PLA General Hospital approved the study.

### 2.2 Demographic and clinical data

At the entry into the study each participant was reviewed to abstract the following variables: gender, age, family history of psoriasis, disease duration. The parameters of MS such as height, weight and BMI, waist circumference, BP and some biochemical index (high density lipoprotein (HDL), triglyeride, fasting blood glucose, serum insulin) were examined. The Psoriasis Area and Severity Index (PASI) scores of baseline and after 10 sections of NB-UVB treatment were valued to demonstrate severity of psoriasis. PASI improvement was calculated by subtracting final PASI to initial PASI and expressed in percentage (%, PASI improvement). There is no missing data about the focused baseline characteristics. MS group was used as the baseline group.

### 2.3 Treatment of NB-UVB

The NB-UVB radiation was given in a Waldmann 7002 cabinet incorporating 40 100-W Philips TL-01 fluorescent lamps (311–313 nm). Patients wore protective UV goggles and men wore genital protection. The energy output was measured with a standard intrinsic UV meter. Initial dose was set as 400 mJ/cm^2^, and the dosage is subsequently increased by 100 mJ/cm^2^ per section. The NB-UVB is administered once every other 1 day. After 10 sections of phototherapy for the observational study, the participants were suggested to continue NB-UVB treatment until achieved clearance.

### 2.4 Metabolic syndrome (MS) definition

Study participants were defined as having MS according to definition of IDF (International Diabetes Federation): for a Chinese person to be defined as having MS, he or she must have central obesity (waist circumference ≥90 cm in male and ≥80 cm in female) plus any 2 of 4 additional factors. These 4 factors are:

1) raised TG level: ≥1.7 mmol/L (150 mg/dL),
2) reduced HDL-cholesterol: <1.03 mmol/L (40 mg/dL) in males and <1.29 mmol/L (50 mg/D) in females (or specific treatment for these lipid abnormalities),
3) raised blood pressure (systolic BP (blood pressure) ≥130 or diastolic BP ≥85 mm Hg) (or treatment of previously diagnosed hypertension),
4) raised fasting plasma glucose [FPG ≥5.6 mmol/L (100 mg/dL)] (or previously diagnosed type 2 diabetes).

### 2.5 Serum collection and ELISA (enzyme-linked immunosorbent assay)

Peripheral blood was extracted before the treatment and after 10 sections of NB-UVB treatment. IL-17, TNF-α, IL-6 levels were evaluated by enzyme-linked immunosorbent assay (R&D Systems, shanghai, China).

### 2.6 Statistical analysis

The Student *t* test and Chi-square test were used to compare continuous and categorical variables respectively. We try to examine potential factors linked to NB-UVB outcome by means of univariate and multivariate logistic regression analysis. Data were showed as mean ± SD and percentages. It was assumed that all these statistics was normally distributed. For the definition of treatment goals in psoriasis, the change of PASI from baseline until the time of evaluation (DPASI) was used. After induction and during maintenance therapy, treatment can be continued if reduction in PASI is ≥75%. The treatment regimen should be modified if improvement of PASI is <50%. In a situation where the therapeutic response improved >50% but <75%, as assessed by PASI, therapy should be modified.^[9]^ Therefore, the cutoff applied in present was 50%. Shapiro–Wilks normality test was used to demonstrate all continuous data were normally distributed. Statistical significance was set at *P*-value ≤.05. Statistics analysis was performed by SAS Statistical Software 9.1.3.

## 3 Result

The demographic and clinical features of the study population are summarized in Table 1. According to the criteria of MS of IDF, there were 13 participants diagnosed as psoriatic patients with MS, the other 42 as psoriatic patients without MS. Age and gender were not significantly different among the 2 groups (*P* = .161 and .788, respectively), and the median values for the baseline PASI score and the disease duration were also similar in group of with MS and group of without MS (*P* = .691 and *P* = .663, respectively). The components of the MS in group of MS were significantly different compared with those in group of without MS (Table 1).

**Table 1:**
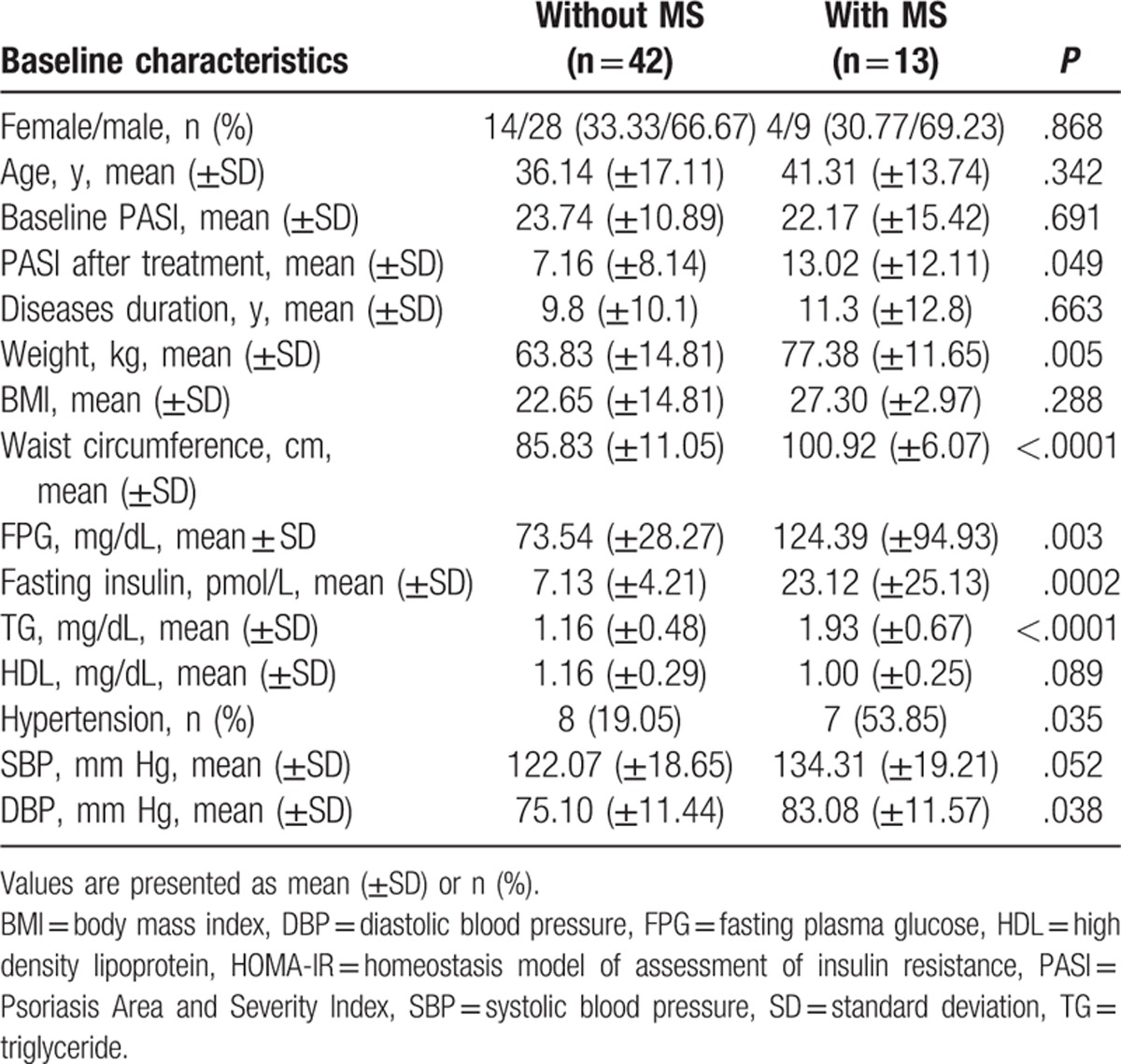
Clinical characteristics of patients with psoriasis vulgaris with metabolic syndrome (MS) and without MS.

### 3.1 Effect of metabolic syndrome on outcome of NB-UVB

**Figure 1:**
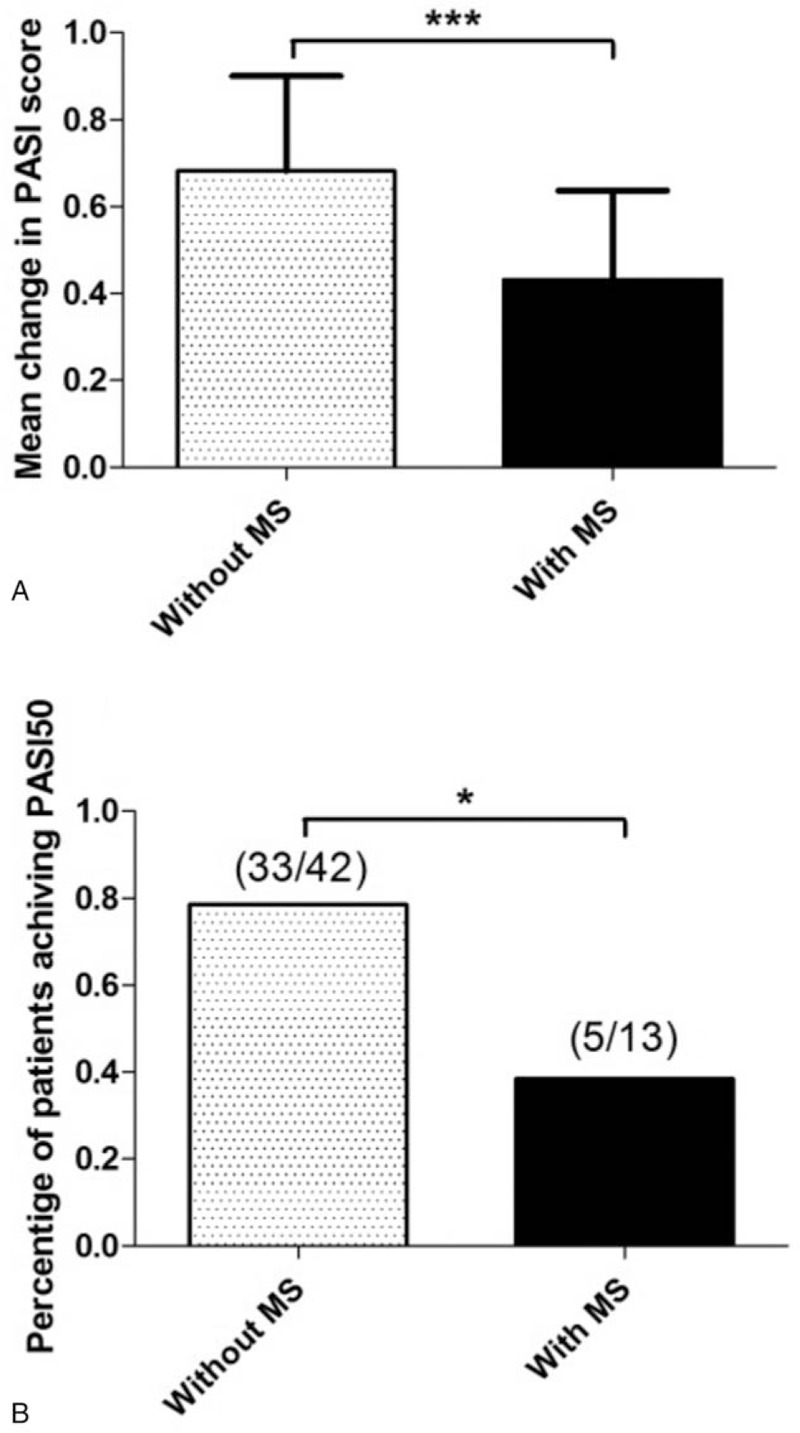
Effect of MS on NB-UVB outcome. Mean change in PASI (A) and percentage of patients achieving 50% reduction in PASI (B) after 10 sessions of NB-UVB treatment in the groups without and with MS from baseline.

Significant PASI improvement was observed in psoriatic patients without MS after 10 sections of phototherapy, while patients with MS showed a less improvement (0.70 vs 0.40, *P* < .001) (Fig. 1A). There was statistically significant difference in percentage of patients achieving 50% reduction in PASI scores between the 2 groups (33/42 vs 5/13, *P* = .0168) (Fig. 1B). Multivariate logistic regression analysis showed MS was an independent factor that affecting the treatment of NB-UVB, that is whether achieving 50% reduction in PASI scores (*P* = .03) (Table 2).

**Table 2:**
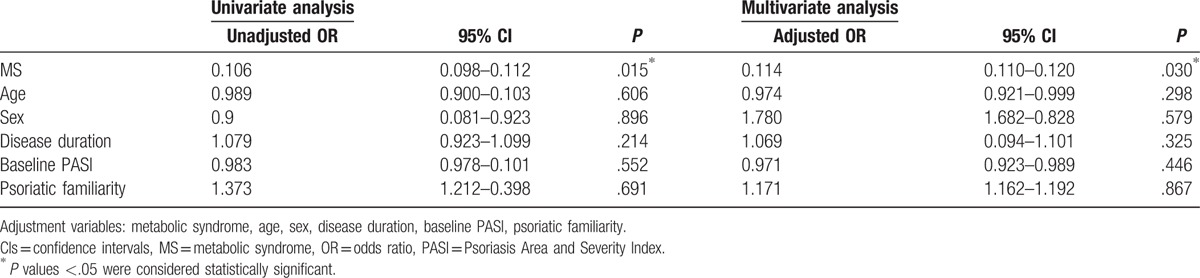
Univariate and multivariate logistic regression analysis of potential factors linked to NB-UVB outcome.

### 3.2 Systemic inflammation

**Figure 2:**
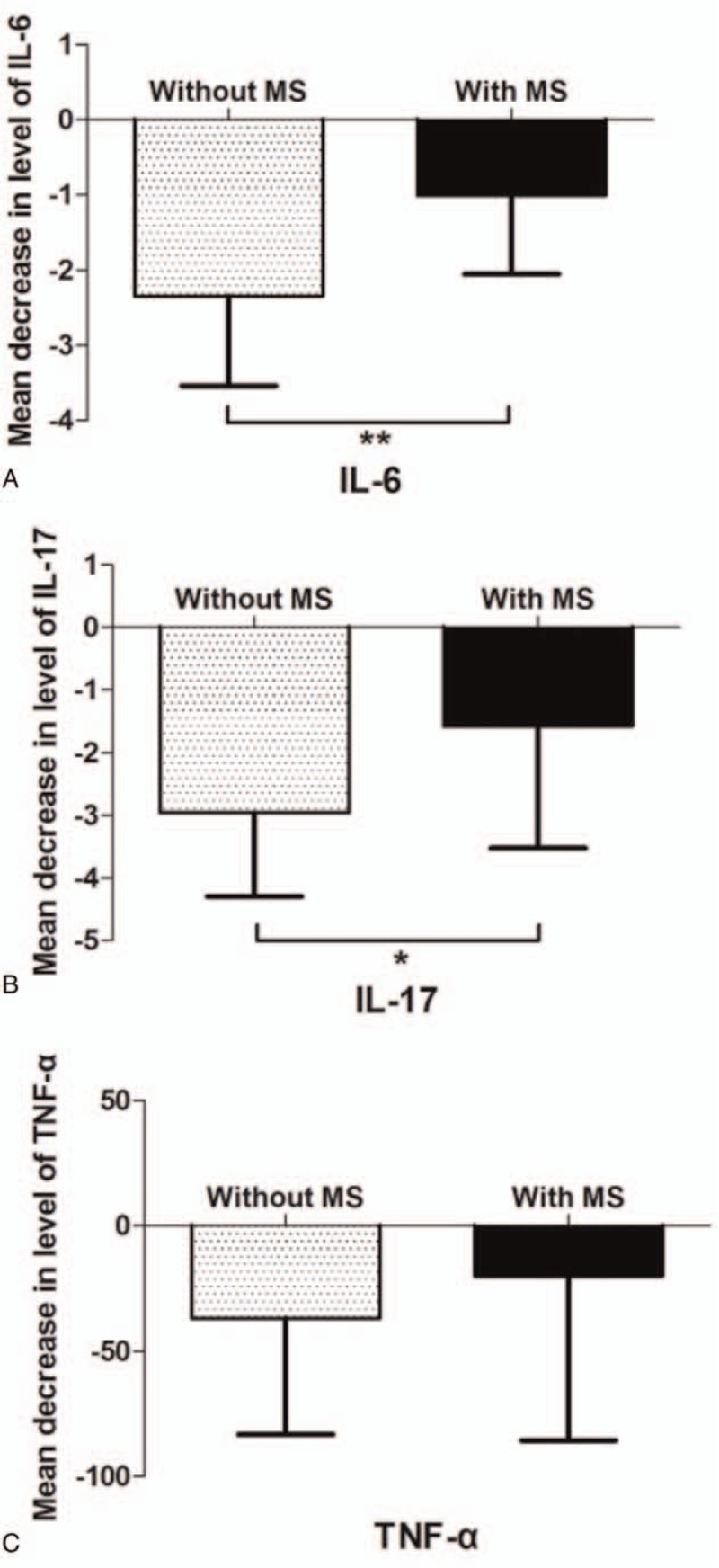
Effect of MS on change of systemic inflammation. Mean decrease in levels of IL-6 (A), IL-17 (B), and TNF-α (C) after 10 sessions of NB-UVB treatment in the groups without and with MS from baseline.

To investigate whether the difference of response to NB-UVB treatment between the 2 groups associates with systemic inflammation, we measured the serum level of IL-6, IL-17, TNF-α before and after 10 sections of NB-UVB treatment respectively. Psoriatic patients with MS showed a much less reduction of serum levels of IL-6 (−2.345 ± 1.188 vs −1.005 ± 1.049) (Fig. 2A) and (−2.959 ± 1.334 vs −1.579 ± 1.943) IL-17 (Fig. 2B) than patients without MS (*P* = .0025 and *P* = .0161). The reduction of TNF-α in group of with MS was lower than in group of without MS, but there existed no statistical significance (−36.87 ± 46.35 vs −20.01 ± 65.54) (*P* = .3743) (Fig. 2C). Multivariate logistic regression analysis showed IL-6 and IL-17 experienced significant reductions in response to the treatment of NB-UVB (*P* = .025, *P* = .012, respectively) (Table 3).

**Table 3:**
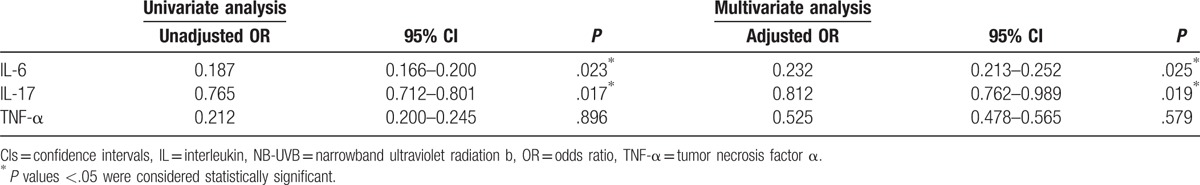
Univariate and multivariate logistic regression analysis of the changing level of IL-6, IL-17, TNF-α linked to NB-UVB outcome.

## 4 Discussion

In present study, we found significant improvement was observed in psoriatic patients without MS after 10 sections of phototherapy in comparison to patients with MS. In addition, our results revealed psoriatic patients with MS showed a much less reduction of systemic biomarkers (IL-17, TNF-α, IL-6) than patients without MS. Although the specific mechanism has been not clear, we assumed that systemic inflammation may be the link between psoriasis and MS. MS, known as a chronic inflammation state, can produce a number of inflammatory cytokine into serum to make systemic inflammation. Psoriasis patient with MS showed higher level of many inflammatory cytokines in serum, such as CRP, TNF-α, IL-17, which may exacerbate the cutaneous inflammatory state so as to promote the progression of psoriasis. Thus, the amount of reduction in inflammatory cytokines directly reflect the treatment of NB-UVB on results of psoriasis.

Psoriasis is a chronic, immune-mediated, and inflammatory skin disease.^[10,11]^ IL-17 pathway plays an important role in the pathogenesis of psoriasis.^[11]^ Recent studies have shown that psoriasis is not only an inflammatory skin disease, but also a systemic inflammation.^[12–15]^ Serum levels of C-reactive protein (CRP), IL-17, IL-22, and other inflammatory cytokines were greatly increased in patients with psoriasis.^[13,14]^ Previous studies have found that serum levels of inflammatory cytokines are positively associated with the severity of psoriatic lesions.^[14–16]^ In addition to that the extent of inflammatory cytokines in circulating system is negatively correlated with the intervals and frequency of recurrence of psoriasis.^[16]^ A recent study found that the recurrence interval of psoriasis patients with MS was significantly shorter in comparison with patients without MS.^[13,15,16]^ These studies suggest that circulating levels of inflammatory cytokines and MS may play an important role in the pathogenesis of psoriasis.^[16,17]^ Our research showed that MS could affected NB-UVB treatment. We know obesity is a poor predictor of UVB treatment in psoriasis.^[17]^ Although there is no study on whether the response of NB-UVB in psoriasis can be affected by other component of MS, epidemiologic studies have shown that the prevalence of insulin resistance, dyslipidemia, and hypertension is higher in patients with psoriasis we compared with general population.^[15,18]^ Meanwhile, insulin resistance may participate in the development of psoriasis by inducing over-proliferation and defected differentiation of keratinocytes.^[13]^

Narrowband UVB is an effective and widely used treatment for moderate to severe psoriasis.^[10,11]^ The specific explanation of UVB treatment for psoriasis lied in a direct inhibiting of T lymphocytes, dendritic cells, Long Hans cells, keratinocytes which can induce apoptosis and activation of regulatory lymphocytes.^[12]^ Further, studies have shown that UVB treatment can also reduce serum levels of inflammatory cytokines obviously.^[12,16]^ However, there existed a significant difference in the effectiveness of narrow-band ultraviolet B treatment in patients with psoriasis according to our clinical experience. The influencing factors may include skin lesion size, plaque infiltration degree, skin type, mental stress, you name it.^[7,10]^ In present study, we found the serum level of inflammatory cytokines is also such a factor we need to notice.

In general, psoriasis was more likely to occur among patients with MS and they always have more inflammatory cytokines in their bodies. In another word, the severity of psoriasis was associated with levels of inflammatory cytokines. Patients with psoriasis who had MS showed significantly lower reduction in levels of systemic inflammation after UVB treatment than those without MS. This may be explained by this possibility that MS can release more inflammatory factors into the circulation and was known as a systemic inflammation, which exacerbates the development of psoriasis.^[8]^ UVB therapy aimed to reduce the number of inflammatory cytokines quickly. Thus patients with psoriasis and MS need receive more UVB treatment against Th1 lymphocytes, dendritic cells, Langerhans cells, keratinocyte apoptosis, and upregulation of Treg lymphocytes which can induce the proliferation and defect differentiation of keratinocyte to sustain the lesion.

UVB is a common clinical treatment of psoriasis, but patients responses to that differently. MS can be used as a predictor of the efficacy of UVB in the treatment of psoriasis, suggesting that patients may need a longer course of treatment to achieve improved skin lesions. This is the first study to investigate whether MS can affect the efficacy of UVB in the treatment of psoriasis. There is no study regarding the effect of MS on UVB treatment of psoriasis. We realized that patients with MS require a longer course of treatment in order to achieve skin lesions cleared than those without MS. We know that MS is characterized by a combination of centripetal obesity, dyslipidemia, insulin resistance, and metabolic abnormalities such as hypertension and it is very popular at present. There is a necessity to clear related details as possible as we can. We speculate that metabolic abnormalities, that is, high-level inflammatory state, should be actively improved in patients with psoriasis in order to promote the treatment effect of narrow-band UVB. It is of great importance for dermatologist to know the effect of MS on treatment of psoriasis.

There are 2 notable limitations in our study. First, we only selected the serum level of IL-17, TNF-α, IL-6 to represent inflammatory cytokines to clear possible effects of MS on treatment of UVB in patients with psoriasis. Second, on account of a relatively small sample size of patients, the reliability of results we obtained is limited. This situation might have influence on our findings. Elucidating this issue more accurately would need larger sample sizes. Those open the door for the future study.

## 5 Conclusion

Psoriatic patients with MS have poorer improvement in comparison those without MS using NB-UVB treatment. MS was an independent factor that affecting the treatment of NB-UVB. In addition, psoriatic patients with MS showed a much less reduction of these systemic biomarkers than patients without MS. Namely, they may need a longer course of treatment to achieve improved skin lesions.

## Acknowledgment

We thank the patients who participated in this study and the staff involved in this work.
